# Radiation Oncology Active Learning in Undergraduate Medical Education: The Usefulness of Kahoot and TikTok

**DOI:** 10.1007/s13187-025-02583-5

**Published:** 2025-02-19

**Authors:** Irene Zapata-Martínez, Francisca Rius-Diaz, Rocío Lorenzo-Álvarez, Lourdes De la Peña-Fernández

**Affiliations:** 1 Department of Pharmacology and Pediatrics, School of Medicine, University of Málaga, Campus Teatinos, Boulevard Louis Pasteur, 32, 29071 Málaga, Spain; 2 Department of Public Health, School of Medicine, University of Málaga, Málaga, Spain; 3 Ronda School of Nursing, University of Málaga, Málaga, Spain; 4 Department of Emergency and Intensive Care, Axarquía Hospital, Vélez-Málaga, Málaga, Spain; 5 Department of Radiology, School of Medicine, University of Málaga, Málaga, Spain

**Keywords:** Radiation oncology, TikTok, Kahoot, Gamification, Active learning, Academic performance, Medical education

## Abstract

Gamification and social networking can be used as active learning tools, as they have been shown to increase learner motivation. Nevertheless, it is unclear whether they impact students’ knowledge acquisition. This study aimed to share an educational experience for medical students using Kahoot and TikTok as active teaching/learning tools and assessed whether they improve academic performance. A retrospective quasi-experimental study was conducted with 176 students enrolled in radiation oncology in the 2021/22 academic year (control group), who received traditional teaching, and 166 students in the 2022/23 academic year (experimental group), where Kahoot and TikTok were added to traditional teaching. Final exam results of both groups were compared to assess learning differences, as well as those of the experimental group in terms of participation in Kahoot and TikTok and their relationship with the final grade. Comparing the average exam scores of the experimental group to the control group showed an increase of 12.9% in the first group compared to the traditional group (6.02 vs. 7.31 points, *p* = 0.0001). In the experimental group, students who participated in Kahoot or TikTok increased their mean score by 0.54 points (*p* = 0.021) and 1.57 points (*p* = 0.040), respectively, compared to those who did not. A correlated increase in mean test scores was observed based on tool usage: none, Kahoot or TikTok, or both (6.19, 6.88 ± 1.48, and 7.51 ± 1.20 points, *p* = 0.019). Using Kahoot and TikTok as active learning tools for medical students improved academic performance when integrated with traditional methodology.

**Supplementary Information:**

The online version contains supplementary material available at 10.1007/s13187-025-02583-5.

## Introduction

### Background

In recent years, there has been a notable change in university students’ attitudes toward how they receive teaching and their interest in it. Since the COVID-19 pandemic, the educational role of technology and social networks has significantly increased [1, 2]. Medical school may consider digital teaching and learning tools to engage digital-native students, enhancing motivation and learning through interactive technology. [3].

Incorporating gamification or game-based learning techniques offers students an engaging way to participate in the learning process, capturing their attention and enhancing knowledge retention due to its “playful” nature. It also serves as a formative assessment tool by providing feedback on individual learning processes [3].

Among the online game-based educational platforms, Kahoot! stands out. Kahoot is a free learning platform that allows the creation of assessment quizzes where students can respond in real time using their mobile devices. The teacher can then conduct test competitions, encouraging participation and reinforcing student learning. Although not frequently used in university education, studies in medical and other health-related school have shown that it increases students’ motivation [4]. Social networks also combine information and education with entertainment, thus motivating students to engage more in the learning process, promoting collaboration, effective self-learning, and creativity [5]. While popular platforms like Facebook or Twitter focus on texts or still images, limiting information sharing, TikTok offers more versatility, allowing you to create short videos, add visual or musical effects, and even stream them live, making it highly popular between young public [6]. Students also have a very positive attitude toward using TikTok as an educational tool in higher education [7, 8], which can also be used to encourage creative manipulation of information by the student.

Both Kahoot and TikTok can be used as digital learning tools to promote “active learning” through activities that encourage students to “learn by doing,” involving them in the process and fostering deep learning skills [3]. These tools offer an alternative to traditional teaching methods by focusing on the learner, encouraging participation, communication, and self-learning skills [9].

There is a clear relationship between the use of these innovative teaching/learning tools and student satisfaction in terms of engagement, motivation, reinforcement, and learning [10, 11]. However, few studies investigated whether these tools also improve the teaching/learning process and contribute to better academic outcomes in medical education [3].

This is the reason why this study was carried out on students at the medical school of the University of Malaga (Spain). Undergraduate students entering Spanish medical schools require excellent grades in high school and national entrance exams and obtain a Bachelor of Medicine degree after completing 360 ECTS (European Credit Transfer System) over 6 years. Specifically, the Degree in Medicine at the University of Malaga is awarded the international seal of the World Federation for Medical Education (WFME) and has the following curriculum: in the first 2 years, basic subjects are taught (anatomy, physiology, biochemistry) together with clinical subjects (surgical bases, pathology, pharmacology). From the third year onwards, clinical subjects predominate, including radiotherapy. Given the aging population and the increase in cancer cases, medical students need to understand the indications and side effects of radiotherapy. Due to the importance of this knowledge for future doctors, this educational research was contextualized for the students enrolled in it.

This study had two main objectives: to share our educational experience using Kahoot and TikTok as digital teaching–learning tools in a course for medical students in the subject of radiotherapy and to evaluate the effectiveness of the intervention, examining whether its use contributed to improving students’ academic performance.

## Methods

A retrospective quasi-experimental cross-sectional study was conducted on third-year students enrolled in the radiotherapy subject at the School of Medicine, Malaga University. The control group consisted of 176 students enrolled during the 2021/22 academic year who received traditional teaching methodology. The experimental group consisted of 166 students enrolled in the 2022/23 academic year, who received the same methodology, with the same professor, but with the addition of innovative teaching/learning tools. These tools included playing Kahoot after each class and/or participating in collaborative work using TikTok.

### Control Group

In the 2021/22 academic year, students received 19 classroom theory classes (divided into four blocks: radiation protection, radiobiology, technical fundamentals of radiation oncology, and the role of radiotherapy in cancers of the main anatomical locations) and 10 h of hospital practice in the radiation oncology service. The course evaluation was based on a theoretical exam and a hospital internship notebook (90% and 10% of the final score, respectively).

### Experimental Group

In the 2022/23 academic year, in addition to traditional teaching methods, innovative teaching tools were used to promote active learning, continuous student evaluation, and digital skills. Two digital tools were employed: Kahoot in the classroom and TikTok for collaborative work. Participation in both activities was voluntary.

Kahoot was used at the end of each thematic block. Students participated individually in the classroom in Kahoot quizzes designed by the teachers, the aim of which was to consolidate and assess knowledge. Each quiz contained between 10 and 15 questions that explored the key points of each topic in depth. After each question was posed, students answered with their mobile phones, and the correct answer was displayed. Teachers then took the opportunity to ask the students why they thought the other options were false, thus seeking a more consolidated learning experience.

For the TikTok activity, students worked in groups of 3–4 people to create a video summarizing a specific course topic. There was a total of 46 topics that were assigned by lot to each group (e.g., treatment teams in radiotherapy, radiotherapy treatment in head and neck cancer). The videos were requested to be no longer than 10 min, explaining the content of the topic in an educational and creative way, using appropriate and scientific language. For the preparation of the script, in addition to the information explained in class, they were asked to use other sources of scientific content. Each group presented the link to the video and a document with the bibliographical sources consulted in Vancouver format on the university’s Moodle platform. The evaluation was carried out by two professors using a rubric that was previously explained to the students (Online resource 1) and whose evaluation criteria were creativity, content, ability to diffuse, reliability of scientific content, and compliance with time.

For the evaluation, participation in Kahoot, collaborative work in TikTok, and the hospital practice notebook each accounted for 10% of the final mark. The final exam, consisting of a face-to-face multiple-choice test with questions on a wide range of topics covered during the course, accounted for 70% of the final mark.

To determine whether the use of these digital tools improved students’ academic performance, final exam scores were compared between the control and experimental groups and between students in the experimental group who used these tools and those who did not.

The exam was identical in structure and difficulty to the control group’s exam, based on a bank of questions categorized by difficulty from previous exams.

#### Ethical and Legal Considerations

This study received approval from the Ethical Committee of the University of Málaga (CEUMA), code 137/202. All users voluntarily participated in submitting questionnaires and signed informed consent. All data was treated anonymously.

### Statistical Analysis

Descriptive statistics were used for categorical demographic variables, while mean and standard deviation were used for quantitative variables. The Student’s *t*-test compared mean exam scores based on student participation in activities, and ANOVA was used for variables with more than two categories. Data normality and homoscedasticity were tested beforehand. The Bonferroni test was used for multiple comparisons when ANOVA was significant. A significance level of *p* ≤ 0.05 was considered. SPSS V24.0 (IBM Corp., Armonk, NY) was used for all analyses.

## Results

Radiotherapy is a compulsory subject taken in the third year of the medicine degree. In the 2021/22 academic year, 176 students were enrolled, of which 66.3% were female compared to 33.7% male. In 2022/23, 166 students were enrolled, with a female-male ratio of 69.3% and 30.7%, respectively.

### Participation in Kahoot and/or TikTok and Relationship with Final Exam Score in the Experimental Group

Only 151 of the 166 students enrolled (91%) took the final exam. Of these, 108 took at least one Kahoot, scoring an average of 0.54 points higher on the exam than those who did not participate (7.47 points vs. 6.93 points, p = 0.021) (Table 1).

**Table 1:** Average exam score as a function of Kahoot and/or TikTok participation in the experimental group

	*n* (%)	Average exam score 2022/23*	*P value*
Kahoot participation			
No	43 (28.5%)	6.93 ± 1.41	0.021
Yes	108 (71.5%)	7.47 ± 1.24	
Kahoot performed			
0	43 (28.5%)	6.93 ± 1.41	0.007
1	25 (16.6%)	6.87 ± 1.21	
2	19 (12.6%)	7.72 ± 0.99	
3	21 (13.9%)	7.27 ± 1.36	
4	17 (11.3%)	7.58 ± 1.27	
5	26 (17.1%)	7.96 ± 1.14	
Total	151 (100%)	7.32 ± 1.31	
TikTok participation			
No	3 (2%)	5.78 ± 2.01	0.040
Yes	148 (98%)	7.35 ± 1.28	

*Quantitative variables were expressed as mean ± standard deviation

When the number of Kahoot in which the students participated was correlated with the mean exam score, it was observed that those who did not participate in any Kahoot had a lower mean score than those who participated in 2 or more Kahoot and that the difference was found to be significant by ANOVA (p = 0.007) (Table 1).

Subsequently, when performing the Bonferroni test, students who played all 5 Kahoot (n = 26) scored significantly higher than those who played none or only 1 Kahoot (7.96 vs. 6.93 (p = 0.018) and 6.87 (p = 0.035), respectively).

Of the 151 students who took the final exam, 98% participated in the TikTok activity. Despite the small number of students who did not participate (*n* = 3), the mean exam score of those who did participate was significantly higher than these, with a mean of 1.57 points higher (*p* = 0.040) (Table 1).

### Participation in Both Activities and Relationship with Final Exam Score in the Experimental Group

**Fig. 1:**
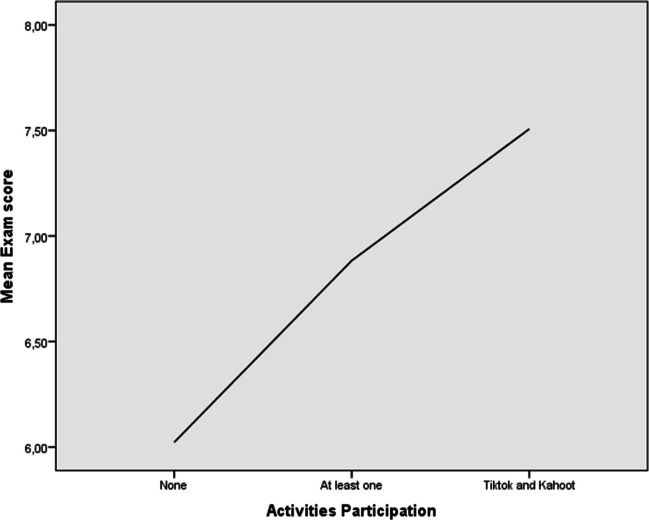
Relationship between the mean exam score and the number of activities performed by students in the experimental group. Notice that the ordinate axis is truncated between 6.0 and 8.0 points out of 10

Of the 151 students who took the final exam, 70.2% (n = 106) participated in both Kahoot and TikTok, 29.2% (n = 44) participated in only one activity, and 1 student participated in none. When comparing the mean of the exam, a significant difference was observed between these groups, and as shown in Fig. 1, there was a linear increase of the score with the number of activities (6.19, 6.88 ± 1.48 and 7.51 ± 1.20 points, when the student did not participate in any activity, in at least one or in both, respectively) (p = 0.019).

### Comparison Between Control and Experimental Groups

The percentage of enrolled students who took the exam was similar between the control and experimental groups (91.5% and 91.0%, respectively).

The students in the experimental group, in whose academic year the use of Kahoot and TikTok was implemented, obtained significantly higher grades than in the control group, with an increase in the mean grade of 12.9% (Table 2) (6.02 vs. 7.31, p = 0.0001).

**Table 2:** Average exam score in the control group (academic course 2021/22) and in the experimental group (2022/23)

	*n*	Average exam score*	*P value*
Experimental group	151	7.32 ± 1.31	0.0001
Control group	161	6.02 ± 1.29	

*Quantitative variables were expressed as mean ± standard deviation

## Discussion

Medical education needs to incorporate evidence-based active learning strategies to enhance student engagement and problem-solving skills [12]. Active learning encompasses different learning methods such as gamification, e-learning, and collaborative learning, where technology and multimedia resources can be used [9]. Gamification and social media have positively impacted student motivation in higher education, and more specially in medical students [3, 7, 10, 13]. However, the impact of these innovative tools on the academic performance has been under-researched. Our study showed that the use of Kahoot and TikTok, compared to traditional methods, significantly improved academic outcomes.

### Influence of Kahoot

In our study, students who participated in Kahoot increased their average final exam score by 5.4%. This result is consistent with other research that used Kahoot in histopathology classrooms [13], as well as in neuroanatomy or histology [14], showing an improvement in the average final exam score of between 2.1 and 9.1% in the groups that used Kahoot compared to a control group. This improvement may be due to the use of this application as a formative assessment tool that helps students regulate their own performance [10].

On the other hand, there are some studies where the use of educational games did not show improvement in academic performance [15]. For example, a study conducted in a single traumatology session compared exam results based on whether or not Kahoot was used at the end of the session [16]. Based on our results and comparison with the published literature, the authors recommend continuous use in the classroom, rather than occasional use, to provide feedback on learning.

### Influence of TikTok

The use of social media is increasingly widespread, with 83% of the population participating in at least one platform [17]. TikTok has been used as a pedagogical tool in other areas of higher education, such as sports science or economics, where it has also been used in the context of collaborative learning and has shown good results in terms of student motivation and creativity [11]. However, there is a lack of evidence on the influence of the use of TikTok on academic results in university education, perhaps because this social network dates back only from August 2018.

In undergraduate medical education, half of students use social media for educational purposes daily [18], which should be used to provide specific training for medical education [6]. In a recent systematic review, the use of social media in this context was associated with improved communication between students and educators, as well as short-term knowledge retention. However, there is little literature evaluating their use as an innovative teaching/learning tool to improve long-term academic outcomes [19].

Our study evaluated the effect of students’ participation in a collaborative basis using the social network TikTok, obtaining in the experimental group an improvement in final scores of 1.57 points compared to students who did not participate. Although we cannot compare our findings with similar studies using TikTok, we found studies in medical education where the use of other social networks as a teaching/learning tool has also shown positive results in student academic performance. A good example was an observational, prospective, and controlled cohort study of geriatrics students using weekly questions on Twitter, with improvements in scores compared to students who did not participate in the social network [20]. Other work with successful evaluation results has used Facebook in anatomy [21], WhatsApp for teaching histology [22], or educational podcasts for learning electroencephalography compared to traditional lectures [23], among others.

On the other hand, educational experiences for medical students using social networks have also been documented without finding benefits in the academic performance of students, such as a randomized controlled trial in which WhatsApp was used in the experimental group of students as a support for teaching pathology, but it was only developed in one subject of the subject. However, they found an increase in student satisfaction with the teaching of the subject [24].

Consequently, although there are few studies that have explored the TikTok social network as a learning tool, our findings are consistent with other studies that have used other social networks as a teaching support tool with increased learning gains.

### Influence of Both Active Learning Tools

Academic results improved significantly, with an increase in mean score of 12.9% when comparing the control group to the experimental group, where Kahoot and TikTok were used as active digital teaching/learning tools.

They also found an improvement in academic performance with the use of gamification and social networks in a “digital literacy” course for medical students [25], as well as in research on pharmacology teaching [9]. In this last study, various active learning strategies were used, such as Kahoot, flipped classroom, and educational videos, compared to a traditional teaching group. However, they did not use combined use of social media.

Our study demonstrates how the combination of Kahoot and the TikTok social network, used as innovation tools for active learning in medical students, was very positive in improving academic results in the subject of radiotherapy. Given these results, the teaching team has continued to use these tools in the following academic years, explaining to students the good results obtained by the classmates who decided to participate in them and motivating them to use it as a tool to improve their academic performance.

Therefore, it seems reasonable to recommend the use of gamification and the use of social networks in the classroom for teaching/learning the subject of radiotherapy in medical schools, as well as to continue exploring the use of these tools in medical education to adapt to the preferences of the new generations with innovative tools that are at the same time facilitators of their learning.

### Limitations

This study has several limitations that are presented below. Student participation in both Kahoot and collaborative work with TikTok was voluntary, and it is unknown if the student’s lack of participation could be related to lower interest in the study. We do not know if there were differences in overall academic results, including other subjects, between the experimental and control groups, although the high qualifications required to access medical studies mean that the population is quite uniform in terms of academic performance. This research was only carried out in one subject and in one institution, so more studies are needed to extrapolate and generalize. In this study, students’ perception of the use of gamification and social networks has not been measured, which may be a good complement for future studies.

Finally, we cannot know which of the two teaching/learning tools improved academic performance, whether it was Kahoot or the social network TikTok, or both, so the authors are considering conducting a second study in which we can compare 3 groups with each other.

## Conclusions

In our study, the use of Kahoot and the social network TikTok in undergraduate medical students in the field of radiotherapy improved their academic performance when integrated with traditional methodology. Kahoot was employed as a tool to offer instant feedback on learning, while the social network TikTok was utilized in collaborative projects to inspire the creative handling of information.

Both tools can be easily implemented in this field of higher education, improving student’s active participation in their knowledge acquisition. Therefore, we consider their use as digital teaching/active learning tools for medical students advisable.

## Supplementary Information

Below is the link to the electronic supplementary material.

Supplementary file1 (PDF 115 KB)

## Data Availability

The data that support the findings of this study are available from the corresponding author (IZM), upon reasonable request.

## Abbreviation

ANOVA: Analysis of variance
