# Supplementary material for: Radiation Oncology Active Learning in Undergraduate Medical Education: The Usefulness of Kahoot and TikTok
Source: J Cancer Educ. 2025 Feb 19;40(6):847–53. doi: 10.1007/s13187-025-02583-5 (PMC12717108; doi:10.1007/s13187-025-02583-5)
Supplement: Supplementary file 1 — Supplementary file1 (PDF 115 KB) [file 13187_2025_2583_MOESM1_ESM.pdf]

Supplementary Material 1: **Rubric for evaluation of collaborative work with TikTok**

Article title: Radiation oncology active learning in undergraduate medical education: the usefull of Kahoot and TikTok

Journal: Journal of Cancer Education

Author names: Zapata-Martínez Irene, Rius-Diaz Francisca, Lorenzo-Álvarez Rocío, De la Peña-Fernández Lourdes.

Corresponding author: Irene Zapata Martínez

Affiliation: Department of Pharmacology and Pediatrics, School of Medicine, University of Málaga. Campus Teatinos, Boulevard Louis Pasteur, 32, 29071 Málaga (Spain).

Phone: 00 34 952 13 16 18

Email: irenezapata365@uma.es

Supplementary Material 1: Rubric for evaluation of collaborative work with TikTok

Evaluation	10	9	8	7	6	5	4	3	2	1	0	Final ratings
**Creativity**	Original work, without losing the teaching/divulgative objective, using innovative methodology.			It does not lose its teaching/dissemination focus, but is limited to mere exposition, with no ideas or creative resources.			Loses teaching/dissemination aspect, reads information				Non-educational/ divulgative, monotonous transmission of content	
**Content**	Clear explanation of scientific content, well argued and well explained.			Good scientific content for the most part, but not all of it.			Content contains errors				The content is not scientific and contains mostly errors	
**Ability to diffuse**	Appropriate use of terminology, oral expression and clarity			Not all terminology and spoken language is appropriate.			In most cases, the correct terminology has not been used.				Overall, there is a lack of focus and clarity on the issue.	
**Reliability of scientific content**	Attachment of scientific bibliographical sources in the correct form			Almost all the content is bibliographically referenced and well documented.			It uses few bibliographical references and expresses them poorly.				No bibliographical references are provided	
**Compliance with time**	Adapts to time			Spends little time or does not distribute the content well within the agreed timeframe			It takes too much time or does not distribute the content in the agreed time.				Completely exceeds of time, or does not reach a minimum	
